# NLRP3 Inflammasome: A New Target for Prevention and Control of Osteoporosis?

**DOI:** 10.3389/fendo.2021.752546

**Published:** 2021-09-27

**Authors:** Na Jiang, Jinyang An, Kuan Yang, Jinjin Liu, Conghui Guan, Chengxu Ma, Xulei Tang

**Affiliations:** ^1^ The First Clinical Medical College of Lanzhou University, Lanzhou, China; ^2^ Department of Endocrinology, The First Hospital of Lanzhou University, Lanzhou, China

**Keywords:** NLRP3, inflammasome, osteoporosis, osteoclasts, osteoblasts

## Abstract

Osteoporosis is a systemic bone metabolism disease that often causes complications, such as fractures, and increases the risk of death. The nucleotide-binding oligomerization domain-like-receptor family pyrin domain-containing 3 (NLRP3) inflammasome is an intracellular multiprotein complex that regulates the maturation and secretion of Caspase-1 dependent proinflammatory cytokines interleukin (IL)-1β and IL-18, mediates inflammation, and induces pyroptosis. The chronic inflammatory microenvironment induced by aging or estrogen deficiency activates the NLRP3 inflammasome, promotes inflammatory factor production, and enhances the inflammatory response. We summarize the related research and demonstrate that the NLRP3 inflammasome plays a vital role in the pathogenesis of osteoporosis by affecting the differentiation of osteoblasts and osteoclasts. IL-1β and IL-18 can accelerate osteoclast differentiation by expanding inflammatory response, and can also inhibit the expression of osteogenic related proteins or transcription factors. *In vivo* and *in vitro* experiments showed that the overexpression of NLRP3 protein was closely related to aggravated bone resorption and osteogenesis deficiency. In addition, abnormal activation of NLRP3 inflammasome can not only produce inflammation, but also lead to pyroptosis and dysfunction of osteoblasts by upregulating the expression of Caspase-1 and gasdermin D (GSDMD). In conclusion, NLRP3 inflammasome overall not only accelerates bone resorption, but also inhibits bone formation, thus increasing the risk of osteoporosis. Thus, this review highlights the recent studies on the function of NLRP3 inflammasome in osteoporosis, provides information on new strategies for managing osteoporosis, and investigates the ideal therapeutic target to treat osteoporosis.

## Introduction

Osteoporosis (OP) is a chronic disease characterized by changes in bone mass, bone microstructure, and fractures and is a causative factor of morbidity and death in senior adults (1). According to the National Health and Nutrition Examination Survey in 2010, more than 50% of seniors in the USA were affected by OP and low bone mass (2). Furthermore, severe medical and social issues caused by OP are accelerated with age (3).

Previous studies have suggested that estrogen is the main hormone regulator of bone metabolism. Estrogen deficiency can not only directly promote osteoclasts differentiation through estrogen receptors on osteoclasts, but also indirectly stimulate nuclear factor kappa-β ligand (RANKL) on osteoblasts, T cells and B cells to promote bone resorption (4). In addition, estrogen deficiency increases osteoblast apoptosis and inhibits osteoblast differentiation by increasing reactive oxygen species (ROS) and nuclear factor kappa-B (NF-κB) pathway, causing a relative deficiency of bone formation (5, 6). As a result, the balance between osteoblasts and osteoclasts is broken while the rate of bone formation cannot keep up with the rate of bone resorption, leading to net bone loss. Therefore, the therapeutic effect of estrogen achieves in OP mainly through four effector cells such as osteoblasts, osteoclasts, osteocytes and T cells (7). Besides the effects of hormones on bone metabolism, chronic inflammation promotes bone loss and the onset of OP (8). The nucleotide-binding oligomerization domain-like-receptor family pyrin domain-containing 3 (NLRP3) inflammasome is an intracellular protein complex that mediates the systemic innate immune response and inflammation. The NLRP3 inflammasome mediates the activation of inflammatory caspase 1 (Caspase-1), interleukin (IL)-1β, and IL-18, causing inflammation and inducing inflammatory cell death.

It has been demonstrated that the abnormal activation of the NLRP3 inflammasome is closely related to multiple metabolic diseases driven by aging and chronic inflammation, such as diabetes, obesity, and gout (9–12). Here, we briefly summarize recent studies on the mechanism of NLRP3 inflammasome in OP, provide information on new strategies for preventing and treating the disease, and investigate the ideal therapeutic target to treat osteoporosis.

## Concept and Structure of NLRP3 Inflammasome

The maturation and activation of IL-1β, a critical molecule involved in inflammation, was known to be mediated by Caspase-1; however, the underlying mechanism remained unclear until the discovery of the NLRP1 inflammasome that suggested the modulation of the process in monocytes (13). The NLRP3 inflammasome, a supramolecular complex concentrated in the cytoplasm, affects innate immunity and inflammation, responds to pathogen- and injury-related signals, and mediates the activity of Caspase-1 and IL-1β (14). Additionally, various sensors are known to assemble into classic inflammasomes such as NLRP1, NLRP3, NLRC4, AIM2, and Pyrin (15).

**Figure 1:**
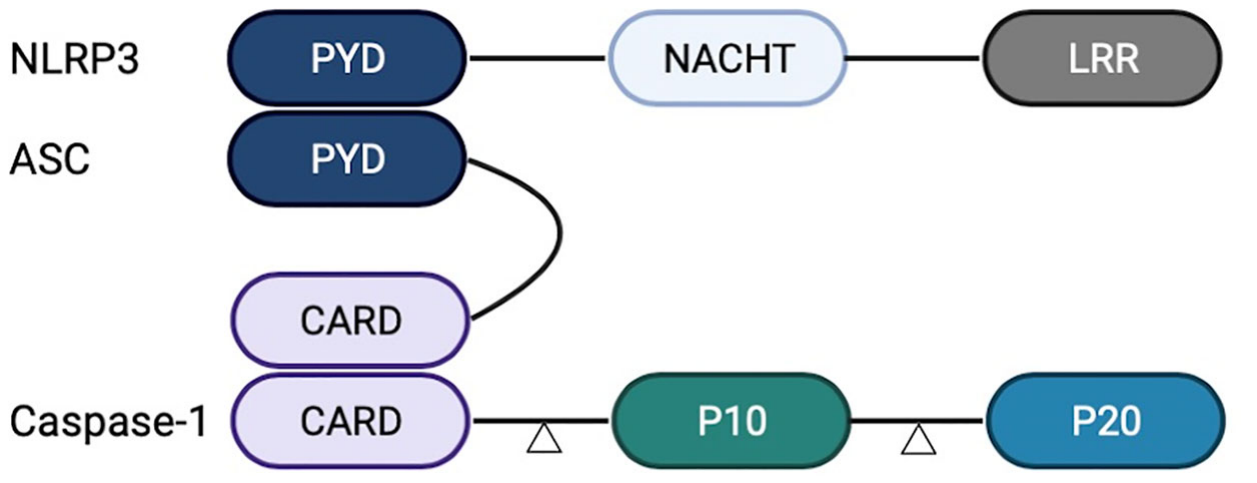
Structure of nucleotide-binding oligomerization domain-like-receptor family pyrin domain-containing 3 (NLRP3) inflammasome. NLRP3 inflammasome comprises a leucine-rich repeat (LRR) domain, an N-terminal Pyrin domain (PYD), and a central adenosine triphosphatase (ATPase) domain known as NACHT. Caspase-1 comprises CARD and two subunits, p10 and p20. NLRP3 and ASC interact with their respective PYDs. ASC and Caspase-1 interact with their respective CARDs.

The NLRP3 inflammasome is mainly composed of a signal sensor component (NLRP3) and an adaptor, apoptosis-associated speck-like protein containing a CARD (ASC). The ASC can recruit the proinflammatory caspase, Caspase-1 (16), or the non-classical caspases, Caspase-4 or Caspase-5 (17). In the inflammasome complex, Caspase-1 is composed of CARD and two subunits, p10 and p20, at the N-terminal. NLRP3 and ASC interact with their respective PYRIN domains (PYD), whereas clustered ASC and Caspase-1 interact with their respective caspase recruitment domains (CARD). The NLRP3 inflammasome acts as the molecular platform for Caspase-1 lysis and activation, and the two subunits form the activated Caspase-1 tetramer (18). The activated Caspase-1 processes the precursors IL-1β and IL-18, resulting in the release of mature cytokines and the induction of the inflammatory response in the extracellular environment (**Figure 1**).

## Regulatory Mechanism of NLRP3 Inflammasome

**Figure 2:**
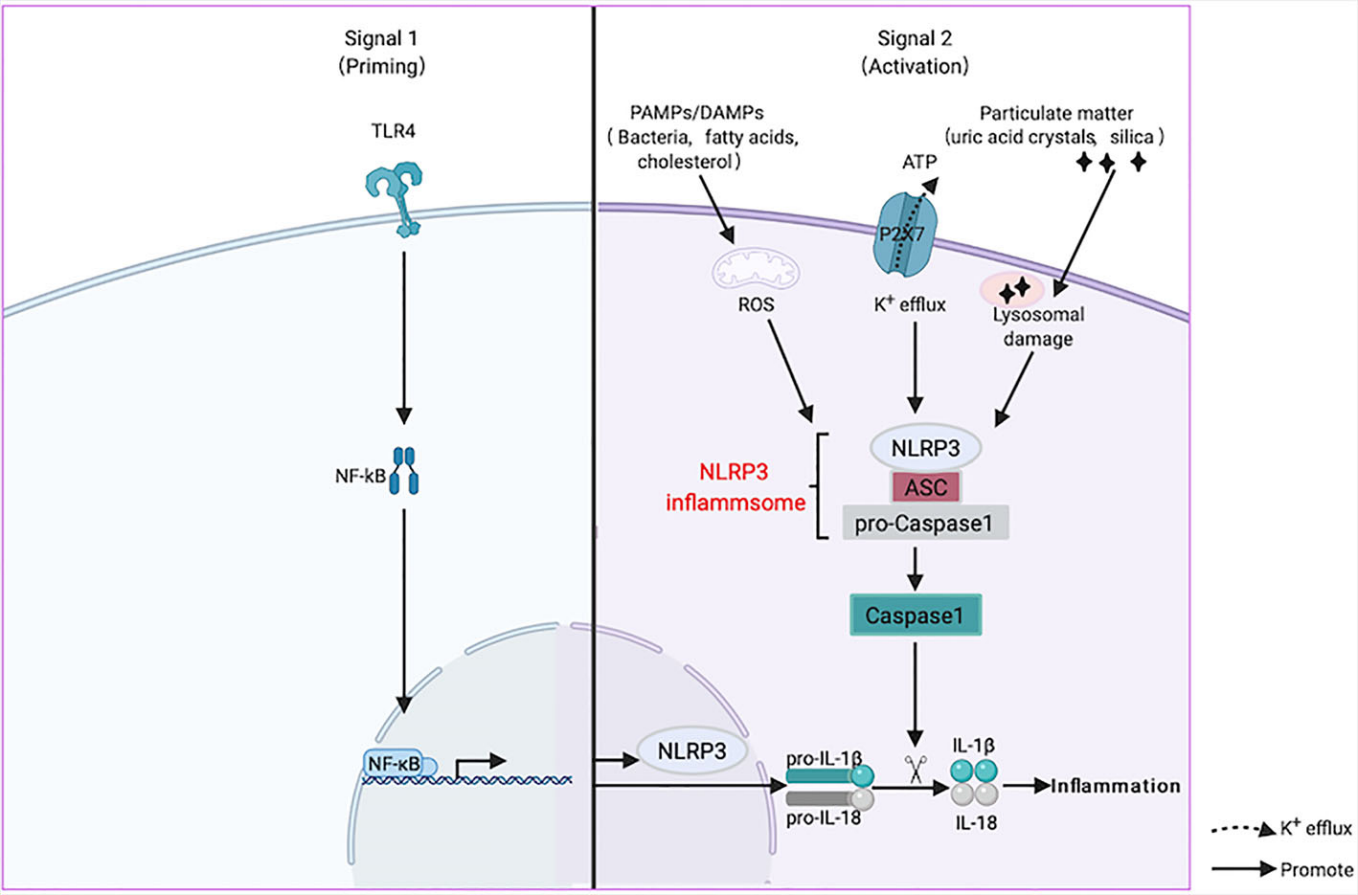
Activation mechanism of nucleotide-binding oligomerization domain-like-receptor family pyrin domain-containing 3 (NLRP3) inflammasome. NLRP3 is activated by two signals when it senses the stimulation of aging or estrogen deficiency through toll like receptors (TLRs). The first priming process (Signal 1) is the expression of NLRP3 and inflammatory factors under the action of the NF-κB transcription factor. Next, it induces uniform downstream host-derived cellular events, including K^+^ efflux, Ca^2+^ efflux, reactive oxygen species (ROS) generation, and lysosomal damage. ASC is an adaptor molecule responsible for connecting NLRP3 and caspase-1 precursors, and then recruits the precursor caspase-1 into an activated form (Signal 2). Activated caspase-1 cleaves the precursors of IL-1β and IL-18 into mature forms and causing inflammation.

The recognition of activation signals by inflammasomes is the first step in inflammatory reactions. NLRP3 recognizes two types of extracellular stimulators, pathogen-associated molecular models (PAMPs), such as bacteria and viruses, and danger-associated molecular models (DAMPs), such as uric acid crystals, saturated fatty acids, and cholesterol (19). Next, it induces uniform downstream host-derived cellular events, including K^+^ efflux, Ca^2+^ efflux, ROS generation, and lysosomal damage (20). The activation of the NLRP3 inflammasome involves priming and activation. It is believed that the transcription of IL-1β and NLRP3, mediated by NF-κB, is the main event caused by the inflammasomes. In addition, post-translational modification of NLRP3, such as phosphorylation induced by JNK1, is a critical event during the initiation process (21). During inflammasome assembly, NLRP3 interacts with a NIMA-related enzyme (NEK7) through the NACHT domain to form large oligomers that form the basis of its activation (22). Finally, NLRP3 recruits Caspase-1 precursor (pro-Caspase-1) through ASC and promotes the processing of Caspase-1 and the subsequent maturation and secretion of the proinflammatory cytokines IL-1β and IL-18, causing inflammation (23) (**Figure 2**).

## NLRP3 Inflammasome Mediated IL-1β and IL-18 in OP

**Figure 3:**
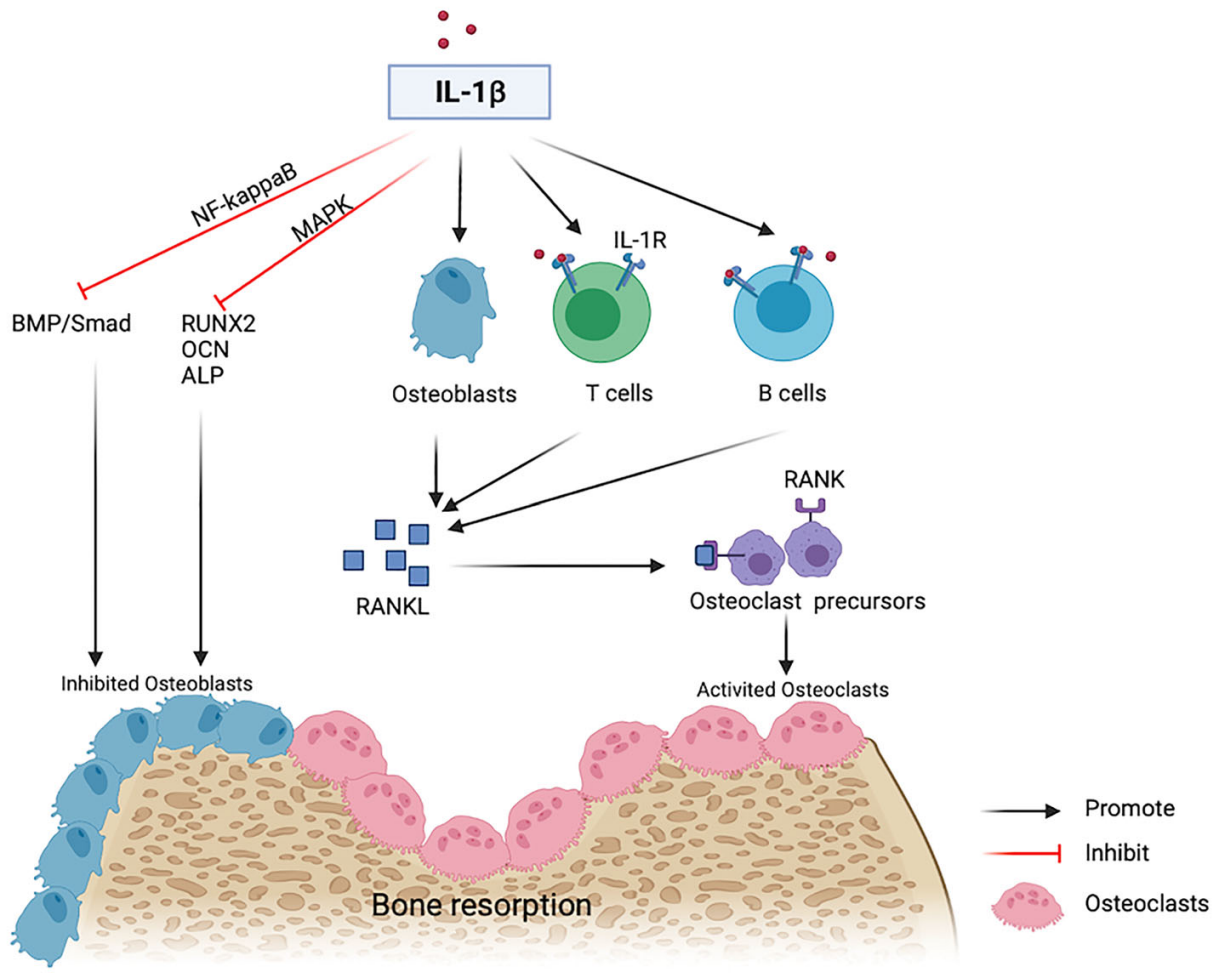
IL-1beta contributes to bone resorption. IL-1β inhibits osteogenic differentiation by inhibiting the BMP/Smad pathway and osteogenic markers including RUNX2, OCN and ALP. IL-1β binds with IL-1R on T cells or B cells and induces the expression of RANKL on osteoblasts and then promotes activated osteoclasts *via* a RANKL-RANK independent mechanism.

Reduced estrogen levels and aging promote low-grade inflammation in the body, and the generated proinflammatory cytokines stimulate OP by affecting the expression and transcription of osteogenic and osteoclastic factors (24, 25). The levels of many inflammatory factors, including IL-1, IL-6, and tumor necrosis factor (TNF)-α, increase during the pathogenesis of OP (26). IL-1β is one of the primary members of the IL-1 family (27) that plays an important role in bone loss following estrogen deficiency (28, 29). A previous study in early postmenopausal women after the discontinuation of estrogen therapy showed that bone resorption is reduced by approximately 50% in subjects randomly receiving anakinra, an IL-1 receptor blocker (30). IL-1β stimulates the expression of Receptor activator of RANKL in osteoblasts or bone marrow mesenchymal stem cells as well as the generation of osteoclasts (25). Besides, IL-1β binds its receptors on T lymphocytes, B lymphocytes, and macrophages, promotes the generation of RANKL, and binds to RANK on osteoclast precursor cells, thus facilitating the differentiation and activation of osteoclasts (31, 32). Therefore, IL-1β is not only an effective bone resorption stimulator but also an effective osteogenic inhibitor. High doses of IL-1β inhibit osteogenic differentiation by activating NF-κB to inhibit the bone morphogenetic protein (BMP)/Smad signal transduction (33). IL-1*β* decreases Runx2 activation and inhibits the osteoblastic differentiation by activating MAPK pathway (34) (**Figure 3**).

IL-18 and IL-1β are closely related since they belong to the same structural family, have similar 3D structures, and their precursors remain inactive until they are cleaved by intracellular Caspase-1 (35). IL-18 promotes osteoclast differentiation through many pathways. Apart from the bone cells, T helper (Th) cells and various other immunocytes are also major factors participating in bone homeostasis (36). Th17 cells release the marker cytokine IL-17 to upregulate RANKL and promote bone resorption (37). Previous studies in ovariectomized (OVX) mice have suggested that increased IL-18 levels in peripheral monocytes stimulate Th17 cells to secrete IL-17, a process conducive to osteoclast differentiation. In addition, co-culture of osteoblasts with CD4^+^ T cells and CD11b^+^ macrophages isolated from OVX mice showed an upregulation of IL-18, activation of NLRP3 inflammasome-related molecules, and inhibition of osteoblasts manifested as reduced expression levels of Wnt-10b, Runt-related transcription factor 2 (Runx-2), and BMP-2 (38). Another *in vivo* study supported the hypothesis that IL-18 participates in the pathogenesis of OP. In OVX mice, the levels of IL-18 binding protein (IL-18BP, a natural specific IL-18 inhibitor) were declined, whereas IL-18BP supplementation markedly decreased the Th17/Treg ratio and proinflammatory cytokines, restoring the microstructure of bone trabeculae. These findings were confirmed in female patients with OP (38). In serum obtained from patients with Cushing’s syndrome, IL-18 and osteocalcin (OCN) levels were negatively correlated (39). Therefore, IL-1β and IL-18 participate in increased inflammation during OP.

## NLRP3 Inflammasome Induces Pyroptosis of Bone Cells in OP

**Figure 4:**
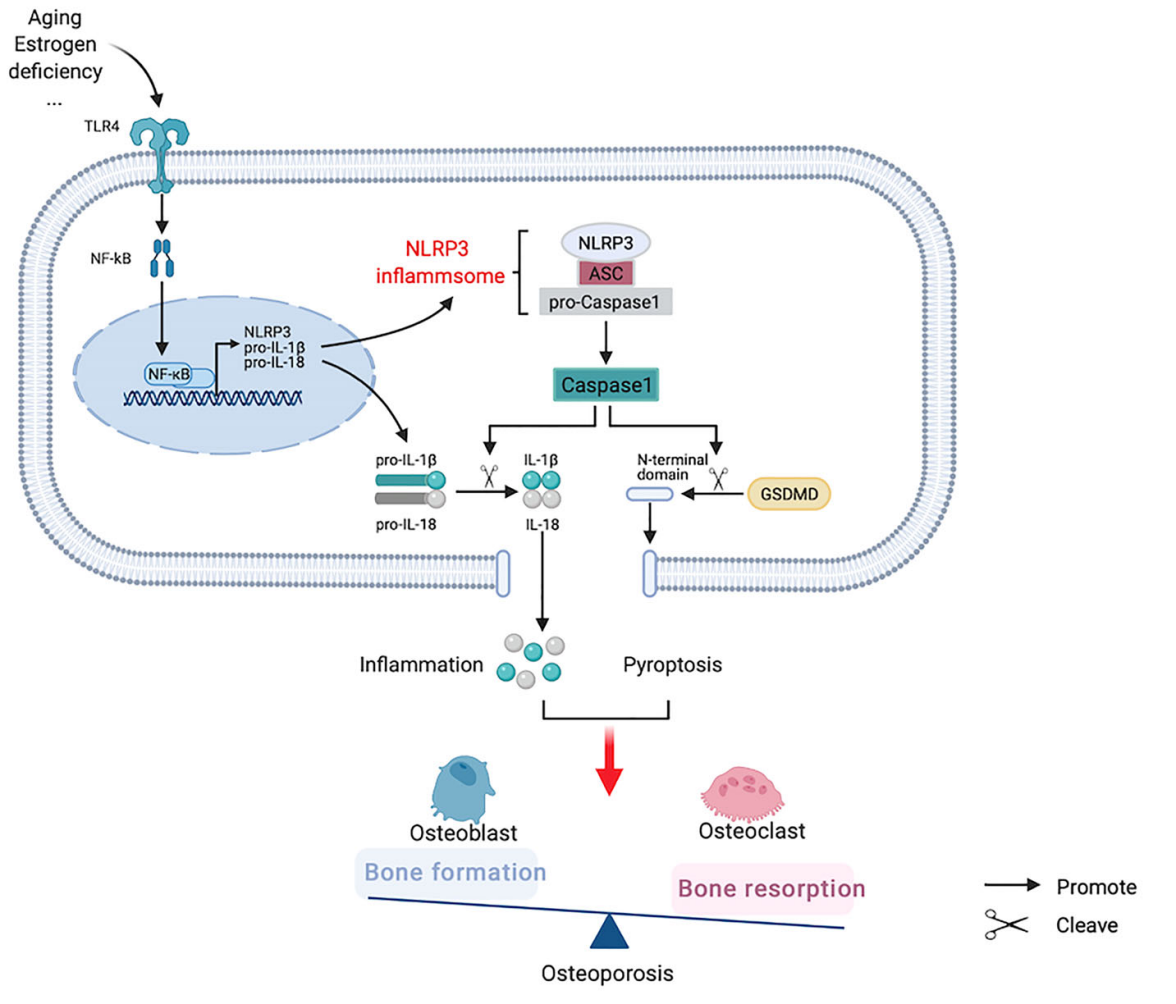
Hypothesized participation of nucleotide-binding oligomerization domain-like-receptor family pyrin domain-containing 3 (NLRP3) inflammasome in the pathogenesis of osteoporosis.

It has been reported that the NLRP3 inflammasome not only aggravates cellular inflammatory response through the Caspase-1/IL-1β/IL-18 activated pathway, but also forms pores on the cell membrane using gasdermin D (GSDMD) as the general substrate, digests the N-terminal domain of GSDMD to bind to the pore of the cell membrane, releases inflammatory mediators, and destructs osmotic pressure, inducing cell swelling, lysis, and death, known as pyroptosis (40). At high glucose concentrations, MC3T3-E1 osteoblasts and Caspase-1 in alveolar bone are activated. Enhanced IL-1β activity increases the expression levels of GSDMD and reduces those of osteogenesis-related proteins, such as p-AKT and β-catenin, whereas Caspase-1 inhibitor can reverse this process (41). In a mouse osteomyelitis model, pyroptosis-related proteins were upregulated, whereas Ac-YVAD-CMK, a specific Caspase-1 inhibitor, not only inhibited the increases of Caspase-1 and GSDMD in mice induced by bacteria, but also helped to restore the osteogenic characteristics (42). Vx765, an inhibitor of Caspase-1, partially reduced bone resorption in apical periodontitis rats, although bone trabecular thickness increased and bone volume did not change significantly. *In vitro* experiments showed that ROS can induce osteoblast pyroptosis and lead to osteoblast dysfunction (43). Tao et al. (44) summarized the relationship between ROS and NLRP3 in OP, considering that ROS is an important component in the pathogenesis of OP, and speculated that it may be a trigger factor for pyroptosis in this pathological process. Therefore, we hypothesize that the NLRP3 inflammasome not only activates the downstream inflammatory factors to participate in OP pathogenesis, but also induces pyroptosis and maintains or aggravates inflammation (**Figure 4**).

## NLRP3 Inflammasome Participates in OP pathogenesis

During the growth and development of bones, the NLRP3 inflammasome and its associated proteins have positive regulatory effects. Compared with NLRP3^+/+^ mice, NLRP3^-/-^ mice have shorter stature, impaired long bone growth, and defective osteoblast differentiation and mineralization (45). ASC is an essential molecule in the osteoblast phenotype. Compared with osteoblasts obtained from wild-type mice, primary osteoblasts with ASC-source knockout have lower levels of osteogenic properties, and thus, tibial defect healing requires a longer period (46). However, over-activation of the NLRP3 inflammasome is related to osteopenia due to aging. A previous study found that NLRP3 is overexpressed in an aging mouse model, whereas its knockout increased the density of the bone cortex and trabecula (47). A humanized NLRP3 mouse strain created by replacing the mouse NLRP3 locus with the human allele associated with the disease developed progressive arthritis and OP, accompanied by granulocyte infiltration and increased IL-1β levels, after attack by injury-related molecular model molecules (48). Recent studies revealed that in an OVX mouse model, the NLRP3 inflammasome components are upregulated in the femoral bone; however, knockdown of NLRP3 notably enhanced the expression of Runx2 and OCN, which are responsible for osteogenic differentiation (49). Therefore, the NLRP3 inflammasome plays a dual role in bone metabolism, but its abnormal activation produces unfavorable effects in OP development (**Table 1**). The NLRP3 inflammasome and its components, IL-1β and IL-18, jointly exert effects in OP, whereas the latter may be regulated by NLRP3 inflammasome to result in inflammatory bone injury.

**Table 1:** The different effects of NLRP3 inflammasome on bone cells.

Effector cells	Effects	Crosstalk pathways	References
Osteoblasts	Decreased cell migration	ROS	Liu SS et al. (43)
	Inhibited the proliferation and differentiation of osteoblasts	p-AKT and β-catenin	Xu L et al. (49)
Osteoclasts	Enhanced bone-resorption capacity of osteoclasts, but inhibited their efferocytosis	ROS/MAPK/NF-κB pathway	An Y et al. (50)
BMSCs	Inhibits osteogenic differentiation and promotes adipogenic differentiation	SIRT1	Wang L et al. (51)

### NLRP3 Inflammasome Inhibits Osteogenesis

Bone mesenchymal stem cells (BMSCs) can differentiate into osteoblasts and adipocytes after stimulation by environmental factors (52). During the development of OP, BMSCs exhibit reduced osteogenic capacity and increased fat-forming capacity, resulting in reduced bone formation and increased bone marrow fat accumulation (53, 54). It has been confirmed that the NLRP3 inflammasome is involved in this process and may affect the differentiation of BMSCs *via* certain active molecules. The group III protein deacetylase, Sirtuin1 (SIRT1), has positive regulatory effects in inhibiting MSC fat formation and promoting bone formation (55). Lipopolysaccharide/palmitic acid (LPS/PA) was previously used to process *in vitro* MSCs by significantly increasing the expression of NLRP3 inflammasome in MSCs and reducing the expression levels of SIRT1; thus, the NLRP3 inflammasome inhibits osteogenic differentiation by inhibiting SIRT1 and promoting the differentiation of MSCs into adipocytes (51). One study showed that osteogenic differentiation is hampered by the NLRP3 inflammasome in MSCs isolated from the human cord blood and treated with LPS/ATP, probably through the inflammasome assembly, since the siRNA of ASC, the critical component of the targeted inflammasome assembly, can reverse the process (56). However, another study showed that the NLRP3 inflammasome does not promote adipogenic differentiation. Any discrepancies between the studies could be attributed to the different stimulants used.

The activated NLRP3 inflammasome contains multiple exogenous or endogenous substances, but none has been confirmed as the activating substances during bone metabolism. A previous study revealed that NLRP3 is expressed in osteoblasts, mediates cell death induced by bacteria, and participates in bone loss during the inflammatory process (57). In OVX mice, the femur protein and NLRP3 in the osteoblasts are highly expressed even when no direct bacterial infection is observed (49). Since various substances can activate the NLRP3 inflammasome during bone metabolism, it is necessary to investigate the mutual regulation mechanism between the metabolites and the NLRP3 inflammasome in OP due to aging or estrogen deficiency.

### NLRP3 Inflammasome Promotes Bone Resorption

Previous *in vivo* experiments have indicated that the NLRP3 inflammasome accelerates bone resorption under multiple bone turnover states (i.e., estrogen deficiency and persistent parathyroid hormone exposure) and that NLRP3 knockout reduces bone loss in many high bone turnover models (58). Similarly, in osteoclasts exposed to high concentrations of glucose and the rat model of diabetic OP, the expression levels of NLRP3 inflammasome and its related proteins are increased, bone density is reduced, and osteoclast markers are increased. Besides, the overactivated NLRP3 inflammasome can be inhibited by exosomes originating from MSCs (59). It has been reported that the initiation step for NLRP3 inflammasome activation mainly depends on the NF-κB pathway. NF-κB is an inflammation regulation signal downstream of Toll-like receptor (TLR) and the classic inflammatory pathway of the immune response. Several studies have confirmed that the NF-κB pathway has significant effects on the growth and maturation of osteoblasts and osteoclasts (6). At the molecular level, exposure to high glucose concentrations upregulated NLRP3 inflammasome expression and this is regulated by ROS/MAPKs/NF-kB. NF-κB inhibitors significantly reduce the expression level of NLRP3 inflammasome and alleviate bone resorption (50). When bone marrow macrophages (BMMs), which are osteoclast precursors, are exposed to bone matrix particles, the NLRP3 inflammasome is activated, resulting in increased NF-kB and MAPK phosphorylation (58). Therefore, the NLRP3 inflammasome and the classic inflammatory pathway mutually interfere and produce joint effects in osteoclast differentiation, which further confirms the critical role of NF-κB in the initiation of the NLRP3 inflammasome.

## Potential Therapeutic Target of NLRP3 Inflammasome in OP

Considering that the NLRP3 inflammasome has dual effects in OP pathogenesis, its regulation may be a novel ideal therapeutic target. Other anti-OP drugs, such as bisphosphonates, parathyroid hormone analogs, and RANKL inhibitors, can only either inhibit bone resorption or promote bone reconstruction to achieve therapeutic effects, and their long-term use has certain safety concerns (1). Previous studies have suggested that bisphosphate, an antiresorptive agent, increases the secretion of NLRP3 dependent IL-1β and induces osteonecrosis in diabetic mice (60). Therefore, combined treatment with an NLRP3 inhibitor might reduce the side effects of bisphosphate, such as jaw necrosis. There are two categories of pharmacological inhibitors for targeting NLRP3 inflammasome: direct inhibitors that directly target NLRP3 protein and some others that are indirect inhibitors, which target constituents of the NLRP3 inflammasome such as Caspase-1, IL-1β and IL-18. Some of the inhibitors that are related to osteoporosis has been reported (**Table 2**).

**Table 2:** Inhibitors of NLRP3 inflammasome related to OP.

Targets	Agents	Benefits	Side effects or limitation	References
NLRP3	MCC950	Reduces age-related bone loss by inhibiting osteoclastogenesis	The effectiveness in bone formation remains to be confirmed	Zhang Y et al. (61)
		Reverses osteogenic dysfunction	*In vitro* experiments only	Liu SS et al. (43)
	CY-09	Reduced bone loss	Osteoarthritis modeling only and needs intra-articular injections	Li Z et al. (62)
	OLT1177	–	–	–
	Glyburide	Expedites diabetes-induced impaired fracture healing	The concentration of anti-inflammatory and anti-hyperglycemia is difficult to balance	Yang X et al. (63)
		Reverses the expression of osteogenic markers and reduces the activation of osteoclasts		Kawahara Y et al. (64)
				Zhu X et al. (42)
	Irisin	Lowers inflammation and suppressed osteoblast apoptosis	Studied in animal models of OP only	Xu L et al. (65)
	Melatonin	Promotes osteoblastogenesis through Wnt/β-catenin pathway	The mechanism mediated through the inhibition of bone resorption is unclear	Xu Lijun et al. (49)
	Dioscin	Inhibits the activation of NLRP3 inflammasome in mouse macrophages and promotes the osteogenesis of mouse pre-osteoblasts	Whether osteogenesis is promoted by inhibiting NLRP3 is unknown	Yin Wei et al. (57)
Caspase-1	Ac-YVAD-CMK	Restores the osteogenic characteristics	Osteomyelitis model only	Zhu X et al. (42)
		Reverses the inhibition of proliferation and differentiation osteoblast resulting from high glucose induced pyroptosis	The effect in bone resorption is unknown	Yang L et al. (41)
	VX765	Partly decreases bone resorption	The effect of vx765 may be limited by dose and duration of the drug	Cheng R et al. (66)
IL-1β	Anakinra	Reduces bone resorption	Blocking cytokines alone cannot completely prevent the increase of bone resorption in estrogen deficiency. Combined blocking may be required	Charatcharoenwitthaya N et al. (30)
	Auranofin	Inhibits osteoclastogenesis and can be orally available	More clinical trial data are needed	Kim H et al. (67)
IL-18	IL-18BP	Inhibits osteoclastogenesis and reduces bone loss.	Humanized IL-18BP toward the treatment of OP remains to be investigated	Mansoori MN et al. (38)

Anakinra, a targeted IL-1β inhibitor, has been successfully applied to treat rheumatoid arthritis (68). A clinical study showed that it is also resistant to bone resorption in postmenopausal women (30). Auranofin, another drug used for the treatment of rheumatoid arthritis, significantly reduces bone loss in OVX mice by suppressing osteoclastogenesis induced by RANKL in BMMs and inhibiting IL-1 expression mediated by inflammasomes (67). The drugs, anakinra and auranofin, successfully regulate the cytokine components of the NLRP3 inflammasome to prevent osteoclast-related OP. Although the drugs alone cannot completely block the inflammation, they can be taken orally rather than through injection. Animal experiments have demonstrated that mouse IL-18BP has obvious anti-OP effects, but humanized IL-18BP needs to be further studied (38).

Novel drugs including natural or synthetic molecules that inhibit the maturation or release of IL-1 family cytokines (i.e., NLRP3 inflammasome or Caspase-1 inhibitor) are currently under development and expected to emerge as a new strategy for the treatment of OP, as confirmed by numerous *in vivo* and *in vitro* experiments. MCC950, an effective and specific inhibitor of the NLRP3 inflammasome, alleviates the inhibition of MG63 osteoblasts mediated by the NLRP3 inflammasome under oxidative stress (43). *In vivo* experiments have suggested that NLRP3 knockout and MCC950 supplementation significantly reduces age-related alveolar bone loss in elderly mice. In addition, MCC950 treatment results in higher bone mineral density and bone volume per tissue volume as well as the reduced formation of tartrate-resistant acidic phosphatase (TRAP)-positive cells, delaying osteoclast differentiation (61). Other targeted NLRP3 inflammasome inhibitors, such as OLT1177 and CY-09, also exhibited good therapeutic properties (69). CY-09 reduced bone loss in osteoarthritis modeling. However, the route of administration is achieved by intra-articular injection in the knee (62). At present, the research on OLT1177 in OP has not been reported.

Glyburide, a drug in clinical use, also known as an NLRP3 inflammasome inhibitor, reversed the expression of osteogenic markers such as collagen I and Runx2 and reduced the abnormal activation of osteoclasts (42). However, in this study, the concentration of glyburide far exceeded the concentration of its application in hypoglycemia, which undoubtedly increased other side effects of the drug. In addition, glyburide accelerated the healing of diabetes induced fracture by inhibiting the production of inflammatory factors (63). In a rat model of periodontitis, glyburide suppressed the activation of Caspase-1 and IL-1β, and oral glyburide significantly the inhibited the number of osteoclasts in alveolar bone (64).

It has been found that many natural and endogenous or exogenous molecules exert anti-inflammatory effects by inhibiting NLRP3 inflammasome activation in OP. Arioz ect (70). have summarized the effect of melatonin, a widely used natural and endogenous molecule, on NLRP3 in a variety of different diseases. *In vivo* experiments have suggested that melatonin inhibits the activation of the NLRP3 inflammasome and improves the inhibition of osteogenic differentiation in OP through the Wnt pathway, which is related to osteogenic differentiation (49). Irisin, another natural molecule, suppressed osteoblast apoptosis and increased the content of ALP in postmenopausal OP rats through the inhibition of NLRP3 inflammasome (65). Dioscin, a plant product, can also inhibit the activation of NLRP3 inflammasome in mouse macrophages and promotes osteogenesis of mouse pre-osteoblasts (57). Therefore, exploring the endogenous and exogenous regulation mechanisms is helpful in improving the understanding of inflammasome activation in the body.

Many studies have focused on the post-transcriptional control of microRNAs (miRNA) based on NLRP3 (71). NLRP3-targeted miRNA is a successful therapeutic method for various diseases, including rheumatoid arthritis and cancer, but little is known about its effectiveness in the treatment of OP (72, 73).

## Summary and Outlook

The expression of NLRP3 inflammasome in bone cells affects osteoblast activation and osteoclast differentiation in OP. In addition, it maintains and aggravates the inflammation of osteoblasts or osteoclasts in OP pathogenesis through its downstream inflammatory factors, IL-1β and IL-18, and the induction of Caspase-1-dependent pyroptosis. The NLRP3 inflammasome is involved in bone metabolism by influencing various active molecules and other classic inflammatory pathways. It is obvious that reduced NLRP3 levels delay OP development, but the underlying mechanism involved needs further research. In order to achieve anti-inflammatory functions, the concentration of some drugs is increased, which also increases the possibility of side effects. Therefore, it is necessary to find more suitable drugs, such as MCC950, to inhibit the progress of OP. Basic and clinical studies that provide references for the prevention and treatment of various metabolic diseases are of the utmost necessity.

## Author Contributions

NJ, JA, and KY contributed equally to this paper. NJ drafted and prepared manuscript. JA, KY, and CM reviewed and edited the manuscript. JL and CG extracted data and constructed figures with software. XT considered for ideas and overall structure of the article. All authors contributed to the article and approved the submitted version.

## Funding

This study was supported by the National Natural Science Foundation of China under grant no. 81370970, and the Science and Technology Support Program of Gansu Province under grants no. 144FKCA075.

## Conflict of Interest

The authors declare that the research was conducted in the absence of any commercial or financial relationships that could be construed as a potential conflict of interest.

## Publisher’s Note

All claims expressed in this article are solely those of the authors and do not necessarily represent those of their affiliated organizations, or those of the publisher, the editors and the reviewers. Any product that may be evaluated in this article, or claim that may be made by its manufacturer, is not guaranteed or endorsed by the publisher.
